# Distinct changes in soybean xylem sap proteome in response to pathogenic and symbiotic microbe interactions

**DOI:** 10.1186/1471-2229-9-119

**Published:** 2009-09-21

**Authors:** Senthil Subramanian, Un-Haing Cho, Carol Keyes, Oliver Yu

**Affiliations:** 1 Donald Danforth Plant Science Center, St Louis, MO, 63132, USA; 2 Plant Science Department, South Dakota State University, Brookings, SD, 57007, USA; 3 Changwon National University, Changwon, Kyoungnam, 641-773, Korea; 4 Maryville University, St Louis, MO, 63141, USA

## Abstract

**Background:**

Plant systemic signaling characterized by the long distance transport of molecules across plant organs involves the xylem and phloem conduits. Root-microbe interactions generate systemic signals that are transported to aerial organs via the xylem sap. We analyzed the xylem sap proteome of soybean seedlings in response to pathogenic and symbiotic interactions to identify systemic signaling proteins and other differentially expressed proteins.

**Results:**

We observed the increase of a serine protease and peroxidase in the xylem sap in response to *Phytophthora sojae *elicitor treatment. The high molecular weight fraction of soybean xylem sap was found to promote the growth of *Neurospora crassa *in vitro at lower concentrations and inhibit growth at higher concentrations. Sap from soybean plants treated with a *P. sojae *elicitor had a significantly higher inhibitory effect than sap from control soybean plants. When soybean seedlings were inoculated with the symbiont *Bradyrhizobium japonicum*, the abundance of a xyloglucan transendoglycosyl transferase protein increased in the xylem sap. However, RNAi-mediated silencing of the corresponding gene did not significantly affect nodulation in soybean hairy root composite plants.

**Conclusion:**

Our study identified a number of sap proteins from soybean that are differentially induced in response to *B. japonicum *and *P. sojae *elicitor treatments and a majority of them were secreted proteins.

## Background

Systemic signaling is crucial for normal plant growth and development. Examples of plant processes that involve systemic signaling include meristem development [1,2], drought stress [3], nutrition monitoring, and microbe interactions [4-6]. Systemic signaling is characterized by the transport of various signal molecules for long (organ to organ) or short distances (cell to cell) to coordinate plant responses to developmental or environmental cues. These signal molecules may include plant hormones [3,7,8], proteins [9], RNAs [10,11], and small molecules [12,13]. Long distance transport of these molecules is mediated by two specialized plant transport systems, xylem and phloem. The xylem stream primarily transports nutrients and water from the roots to the aerial organs of the plant; whereas the phloem stream primarily transports organic compounds resulting from photosynthesis in the leaves to other plant organs. The composition of both xylem and phloem saps are complex [9,14,15] due to the addition of various compounds by different organs either for nutritional or signaling functions. Xylem sap analysis under various biotic and abiotic stress conditions might provide clues about what signaling events occur in response to those stresses. We analyzed the proteome of soybean xylem sap in response to two different soil microbes.

The soybean root and stem rot pathogen *Phytophthora sojae *causes major yield losses throughout the world [16]. *P. sojae *zoospores colonize soybean roots, attach, encyst and penetrate into the host tissue through a germ tube, causing large water-soaked necrotic lesions [17]. Some soybean genotypes can recognize the presence of pathogen-associated molecules, and initiate defense responses including hypersensitive response (HR) cell death [18], leading to resistance against the disease [19]. We recently identified a group of proteins and small phenolic molecules, that are essential for HR cell death and resistance against *P. sojae *in certain soybean lines [20,21]. During HR responses, plants also initiate systemic signals to activate defense responses in distal tissues [22]. These general systemic signals have been characterized in a number of plants beyond soybean [4,5]. For example, systemic increase in the levels of salicylic acid (SA) was observed in tobacco plants infected with mosaic virus [7,8,23]. Other studies have identified pathogenesis-related (PR) proteins and anti-fungal proteins in the xylem sap [24-28]. For example, abundance of a pathogenesis-related protein (PR5) increased in the xylem sap of tomato in response to vascular wilt fungus (*Fusarium oxysporum*) infection [25]. However, it is unclear if the increase in abundance of these proteins in the sap contributes to systemic resistance responses. Additionally, changes in the soybean xylem sap proteome in response to pathogen infection have not been investigated. We chose to examine the proteome composition of soybean xylem sap in response to the wall glucan elicitor (WGE) of *P. sojae*. WGE is a pathogen-associated molecule that elicits HR cell death [16,29,30] in a genotype-independent manner in soybean [20]. We treated soybean seedlings with *P. sojae *WGE to elicit defense responses and assayed changes in xylem sap proteome. We also investigated the antifungal properties of the soybean xylem sap.

While a number of plant-microbe interactions such as *P. sojae *root rot are pathogenic in nature, some are mutualistic/symbiotic in nature. Nitrogen-fixing root nodules result from such a symbiotic interaction between legumes and rhizobia bacteria. Major stages in the development of nodules include surface colonization of root hairs by the rhizobia, transport of rhizobia into inner cortex cells by infection threads, initiation of nodule primordia, differentiation and release of rhizobial bacteroids into nodule primordia cells, nodule cell differentiation and nodule growth [31-33]. Once 'sufficient' amounts of nodule primordia have been initiated, the legume host inhibits additional nodule development. This phenomenon is termed auto-regulation of nodulation [34-37]. Auto-regulation is the result of long distance systemic signaling between the root and the shoot. It is well established that signals from the root dictate the shoot tissue to generate suppressing signals sent back to the root to inhibit further nodulation. Earliest evidence for the control of root nodulation by aerial organs came from grafting studies. For example, the shoot portion of a super-nodulating soybean mutant defective in auto-regulation when grafted onto wild-type roots, caused super-nodulating phenotype in the composite plants [34,38]. Genetic analyses identified a conserved leucine-rich repeat receptor kinase that mediates this long-distance systemic signaling [35-37,39]. However, other signaling components of this pathway are yet to be identified. Some physiological evidence suggested that auxin, brassinolides, plant nitrogen status and/or other plant molecules might mediate this long distance regulation of nodulation [40-42]. It is obvious that systemic signaling is an integral part of legume symbiotic nodulation. Xylem sap is the primary conduit that transports molecules from the root to the shoot. We examined the proteome of the xylem sap to examine and identify polypeptide signals if any that are induced in response to *B. japonicum *treatment. A comprehensive xylem sap proteome analysis reported recently served as our reference [43]. The study analyzed xylem sap proteome at later stages of nodulation and therefore did not analyze the initial signaling [43]. We examined an earlier time point (8 h) and also compared changes in sap composition between symbiotic and pathogenic interactions.

In the present study, we isolated xylem sap from soybean seedlings either inoculated with *B. japonicum *cells or treated with *P. sojae *WGE for 8 h. Separation and analysis of proteins larger than 1 kD by 2D gel electrophoresis revealed the complexity of the soybean xylem sap proteome. We observed many changes in the composition of the sap proteome in response to the above treatments. Our results indicate that soybean xylem sap might contain some proteins that support fungal growth and others that inhibit fungal growth. Xylem sap collected from *P. sojae *WGE-treated plants possessed increased potency towards inhibition of fungal growth.

## Results and Discussion

### Collection of soybean xylem sap

We analyzed the xylem sap proteome of soybean plants in an attempt to identify proteins potentially involved in systemic signaling during two different plant-microbe interactions: response to the fungal elicitor *P. sojae *WGE and symbiotic association with *B. japonicum*. Following 8 h of treatment, we decapitated soybean hypocotyls and collected xylem sap generated by root pressure for up to one hour by pipetting (See methods). Each plant yielded ~120 μl of sap by this method with a protein concentration of ~8 μg mL^-1^. This concentration is comparable to reports in corn (12 μg mL^-1^; [28]), squash (19 μg mL^-1^; [44]) and soybean (10-12 μg mL^-1^; [43]), but was much lower than approximately 100 μg mL^-1 ^total protein concentration in the xylem sap of broccoli, rape, pumpkin, and cucumber [14]. Collection of sap was performed early in the morning as the flow rate of sap was the highest at that time (~120 μL h^-1 ^per plant). The sap continued to flow for up to four hours after decapitation albeit at much lower flow rates. Indeed, a recent publication reported collection of soybean xylem sap by root pressure for up to 28 h. They observed no detectable change in sap composition during this time period [43].

### Two-dimensional gel electrophoresis

**Figure 1:**
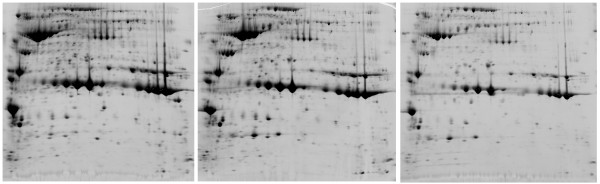
**Reproducibility of 2-D gel electrophoretic separation of xylem sap proteins**. Each gel represents one biological replicate collected independently from untreated soybean plants. At least three biological replicates were used in comparative analyses.

**Figure 2:**
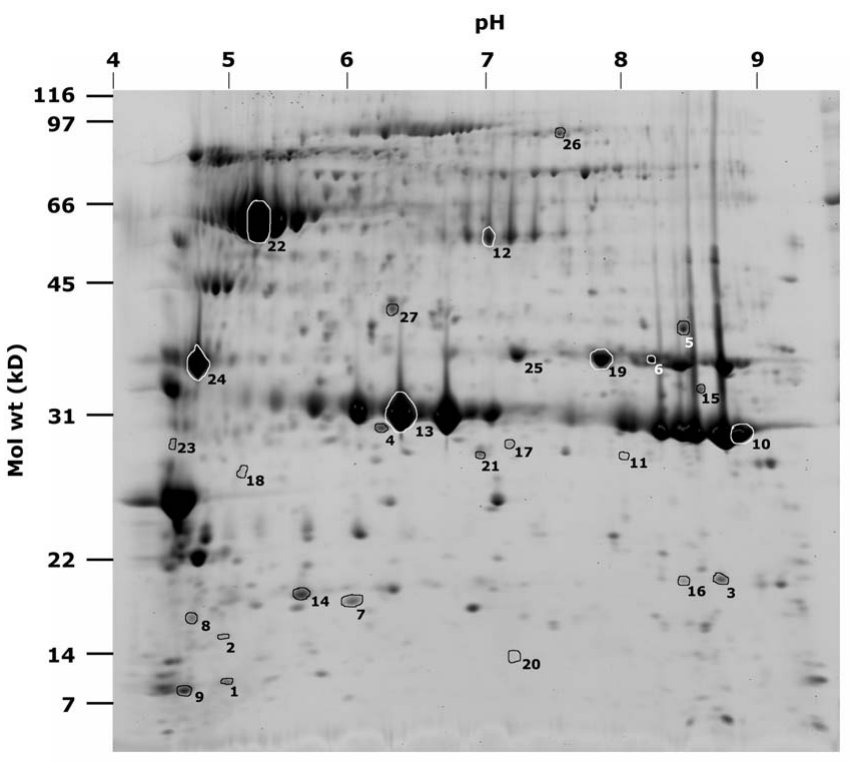
**2-D gel electrophoresis of soybean xylem sap proteins**. Approximate values for the pH gradient are indicated on the top axis and molecular masses on the left axis. Spots that were excised and identified by mass spectrometry are labeled with spot ID numbers listed in Table 1.

The xylem sap was prepared for proteome analysis by excluding molecules smaller than 1 kD through centrifugal filtration and by concentrating proteins through trichloroaceticacid precipitation. Concentrated xylem sap proteins were then separated by 2D gel electrophoresis. Proteins spots were detected by Sypro staining and reproducibility was confirmed by analyzing three independently collected samples i.e. biological repeats (Figure 1). Samples from different biological repeats were reproducible and generated almost identical spot patterns. The most variability was noticed at the smaller molecular weight range (Figure 1). This is perhaps due to the poorer detection of these spots by Sypro staining. Where total protein contents were slightly different between gels (primarily due to variations in efficiency of sample uptake by isoelectric focusing strips), spots volumes were proportional to total volume of all the spots (data not shown). Therefore, we were able to normalize the volume of spots using the total spot volume on the gel. Automatic detection by the Phoretix program enabled visualization of ~600 protein spots. The smallest protein detected was about 8 kD and a number of spots detected were over 200 kD (Figure 2). Spots were detected through a range of pI values from 3 to 10. A majority of the xylem sap proteins migrated in the acidic range. The pH of the soybean xylem sap was ~5.6.

### Protein identification by mass-spectrometry

In two different experiments (using Phoretix software), each with three independent 2D gels per treatment, the proteomic pattern of untreated xylem sap was compared to sap from plants treated with either *P. sojae *elicitor or *B. japonicum*. Normalized spot volumes between samples were compared to identify spots with significantly different volumes (P < 0.05; Student's t-test) between control and treated samples. Spots deemed different between the different treatments and some control spots were excised and identified by nano ESI-MS/MS. Of the 30 spots chosen for identification, only 26 spots that had at least two or more matching peptides from candidates in the database were scored as positive (Table 1). All of these had corresponding tentative contig sequences present in the soybean TIGR transcript assembly (TA) database . Database annotations and the corresponding information from Uniprot database  were used to identify the protein and to estimate its molecular weight and pI values (Table 1, Figure 2). For a number of spots, corresponding Uniprot entries from soybean or wild soybean were identified. For the other spots, the identified Uniprot entries were from other plant species. We compared the observed molecular weight and pI with the theoretical values from the database (Table 1). For most of the spots that had a corresponding soybean entry in Uniprot, the observed pI and molecular weights closely matched that of the theoretical values. Deviations from the theoretical values were only observed for spots with corresponding entries from other species. This is expected due to sequence variations from species to species or multiple isoforms in the same species. Indeed, we observed a number of proteins that had multiple isoforms of the same molecular weight (e.g. β-amylase, spot no. 22; putative subtilisin precursor, spot no. 26; stem glycoprotein, spot nos. 10 and 13; Figure 2). Another possible reason for deviation from expected theoretical values is protein fragmentation. This seemed to be the case for some proteins in our study (e.g. Trypsin inhibitor, spot nos. 1,9).

**Table 1:** Identification of protein spots deemed different between sap from control and *P. sojae *elicitor or *B. japonicum *treated plants

**Spot No**	**Annotation [organism]**	**TIGR** **TA ID**	**Number of peptides matched**	**MASCOT Score**	**Observed** **pI/Theoretical pI**	**Observed MW/Theoretical MW (kD)**	**Signal** **peptide**	**Response to treatment^a^**
1, 2, 7, 8	Trypsin inhibitor A precursor [Glycine max (Soybean)]	TA42304_3847	5	147	5.0/4.6	10.9/20.1	Yes	
3	Stem 31 kDa glycoprotein precursor [Glycine max (Soybean)]	AI736946	4	144	8.5/8.5	15.7/29.3	Likely	
4	Stem 31 kDa glycoprotein precursor [Glycine max (Soybean)]	TA42145_3847	10	385	6.1/5.9	27.6/25.3	Yes	
5	Gamma-glutamyl hydrolase precursor [Glycine max (Soybean)]	TA43569_3847	7	482	7.9/6.3	34.6/35.3	Yes	Bj--
6	CPRD14 protein -- Cinnamoyl alcohol dehydrogenase [Vigna unguiculata (cowpea)]	TA46458_3847	10	537	7.9/5.9	34.6/35.8	No	
9	Trypsin inhibitor A precursor [Glycine max (Soybean)]	TA42304_3847	5	247	4.5/4.6	10.4/20.1	Yes	
10, 11, 23	Stem 31 kDa glycoprotein precursor [Glycine max (Soybean)]	TA41989_3847	13	705	8.7/8.5	27.0/29.3	Likely	
12	Acid phosphatase precursor [Phaseolus vulgaris (Kidney bean) (French bean)]	TA42402_3847	8	392	7.0/5.8	63.6/49.7	Yes	Bj--
13, 14	Stem 31 kDa glycoprotein precursor [Glycine max (Soybean)]	TA42145_3847	15	1042	6.4/5.9	30.9/25.3	Yes	
15	Xyloglucan endotransglycosylase [Malus domestica (Apple) (Malus sylvestris)]	TA43786_3847	5	205	8.4/6.8	32.3/31.9	No	Bj +++
16	Cyclophilin [Kandelia candel]	TA43384_3847	3	89	7.0/8.7	15.3/18.3	No	Bj++
17	Cytosolic malate dehydrogenase [Glycine max (Soybean)]	TA43756_3847	4	173	6.8/6.3	24.6/35.5	No	Bj++
18	20S proteasome beta subunit C-1 [Arabidopsis thaliana]	TA46641_3847	2	87	5.1/5.3	21.0/22.8	No	Bj++
19	Gamma-glutamyl hydrolase precursor [Glycine max (Soybean)]	TA43569_3847	6	315	7.6/6.1	37.8/37.7	Yes	
20	Cationic peroxidase 1, putative, expressed [Oryza sativa (japonica cultivar-group)]	TA64044_3847	2	136	7.0/5.34	10.9/27.0	Yes	Bj--
21, 22	Beta-amylase [Glycine max (Soybean)]	TA44402_3847	4	203	6.8/5.4	24.6/56.2	No	
24	Putative globulin/legumin [Arabidopsis thaliana]	TA42556_3847	4	213	4.7/5.8	37.8/38.3	No	
25	Cationic peroxidase 1 precursor [Arachis hypogaea (Peanut)]	TA59475_3847	2	134	7.0/8.1	38.7/31.2	Yes	Ps++
26	Putative subtilisin precursor [Glycine max (Soybean)]	TA41917_3847	3	275	7.3/6.6	97.8/80.0	Yes	Ps++

^a ^Increase (+) or decrease (-) of spot intensity in response to *B. japonicum *or *P. sojae *elicitor treatment is indicated.

### Origin of sap proteins

The protein sequence was also used to determine if a potential signal peptide sequence was present to allow the proteins to be trafficked to extra cellular spaces (Table 1). A majority of the spots identified (65%) had putative signal peptide sequences. This indicated that most of these proteins reached the xylem sap through the secretory pathway. The remaining proteins could be from the developing xylem parenchyma cells themselves. Indeed, most of the proteins that lacked a potential signal sequence in the precursor were putative cell wall proteins (e.g. xyloglucan endotransglycosylase, spot no. 15). The abundance of many of these proteins was consistently lower in most samples again pointing to a xylem cell cytoplasmic origin (cyclophilin, spot no. 16; Figure 2). We did not find typical phloem sap proteins, such as PP1 protein among the identified spots. However, death of xylem cells and/or differentiation or small levels of contamination from phloem sap are possible explanations for the release of non-secretory proteins in to the sap [28].

### Xylem sap composition in response to *P. sojae *elicitor

The soybean root rot pathogen *P. sojae *colonizes cortex, epidermal and vascular tissues in the root [17]. It is not known if any protein signals are transmitted in response to pathogenic infection through the xylem sap. Therefore, we examined changes in the xylem sap proteome in response to *P. sojae *WGE, a glucan elicitor derived from fungal cell walls. WGE initiates pathogen responses in soybean roots and this response is not race-specific [20]. We applied WGE suspension (10 μg mL^-1^) to the roots of soybean plants and assayed xylem sap proteome composition 8 h post treatment. Interestingly, the total sap protein content increased from 7.76 ± 1.21 μg mL^-1 ^to 11.12 ± 0.97 μg mL^-1 ^upon WGE treatment. Changes in protein content of xylem sap and plant tissues have been observed in response to drought stress [15] and pathogen infection [25]. However, it is not known if this is due to increase in xylem loading of proteins or a mere reduction in xylem water content leading to increased protein concentration.

**Figure 3:**
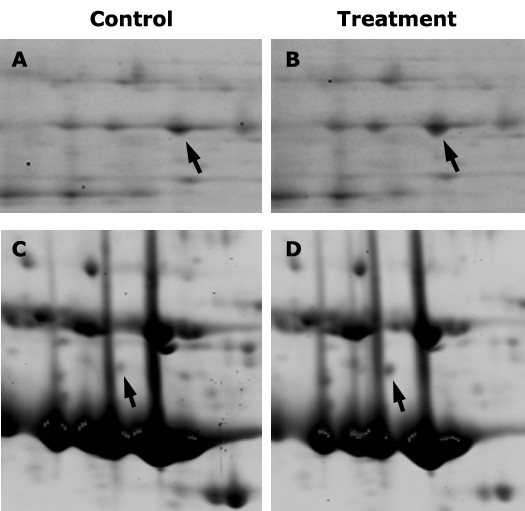
**Portions of 2-D gels showing spots (arrows) that were differentially abundant between xylem saps of control (A, C) and elicitor (B) or *B. japonicum*-treated (D) soybean plants**. Arrows in A & B indicate spot no. 26 (putative subtilisin) and arrows in C & D indicate spot no. 15 (XET).

When the composition was examined by 2D gel electrophoresis, we observed that some protein spots were varied in abundance between sap from WGE-treated and untreated plants. Spots identified as a subtilisin-like serine protease (spot no. 26) and a peroxidase (spot no. 25) increased in response to WGE treatment (Figure 3A, B). Both these proteins have been associated with programmed cell death (PCD) [45-48]. Plants commonly utilize PCD as a defense mechanism against pathogenic microbes [18,49] and PCD plays a role in xylem differentiation as well [45]. PCD responses have been observed in soybean roots in response to both *P. sojae *infection and WGE treatment [20]. Additionally, increase in peroxidase activity has also been observed by histo-biochemical staining assays [20]. Therefore, it is not surprising that we observed proteomic evidence for PCD in response to WGE treatment. What is interesting is the observation of these proteins in the xylem sap. Oxidative burst and peroxidase activity have been observed in roots undergoing PCD including in tissues distal to the site of infection, indicating that distal PCD might be induced by systemic signals [20]. It is possible that peroxidases in the xylem sap could be the result of such a systemic oxidative burst in response to WGE treatment. It still remains to be seen if the subtilisin and peroxidase in the sap play any role in PCD.

Alternatively, certain peroxidases are also known to play a role in lignin biosynthesis [50]. Lignin is a phenolic polymer providing structural fortification to plant cell walls. Lignification is commonly observed in response to mechanical damage or pathogen infection [51]. Therefore, it is also possible that peroxidase induction in the xylem sap could be aimed at lignification as a consequence of detecting a pathogenic molecule. We cannot exclude the possibility that the presence of these proteins in the xylem sap could be due to the involvement of PCD in xylem differentiation. Previous studies have identified a number of pathogenesis-related (PR) proteins in the xylem sap (e.g. PR1a, PR5; [25,52]). We did not identify any PR proteins that were differentially abundant in our study. This could be because we analyzed early changes in the xylem sap proteome in response to elicitor treatment where as the other studies analyzed late responses during disease development in response to compatible pathogen infection. Nevertheless our observation of increase in proteins associated with PCD is consistent with physiological responses to WGE treatment of soybean roots.

### Effect of xylem sap proteins on fungal growth

**Figure 4:**
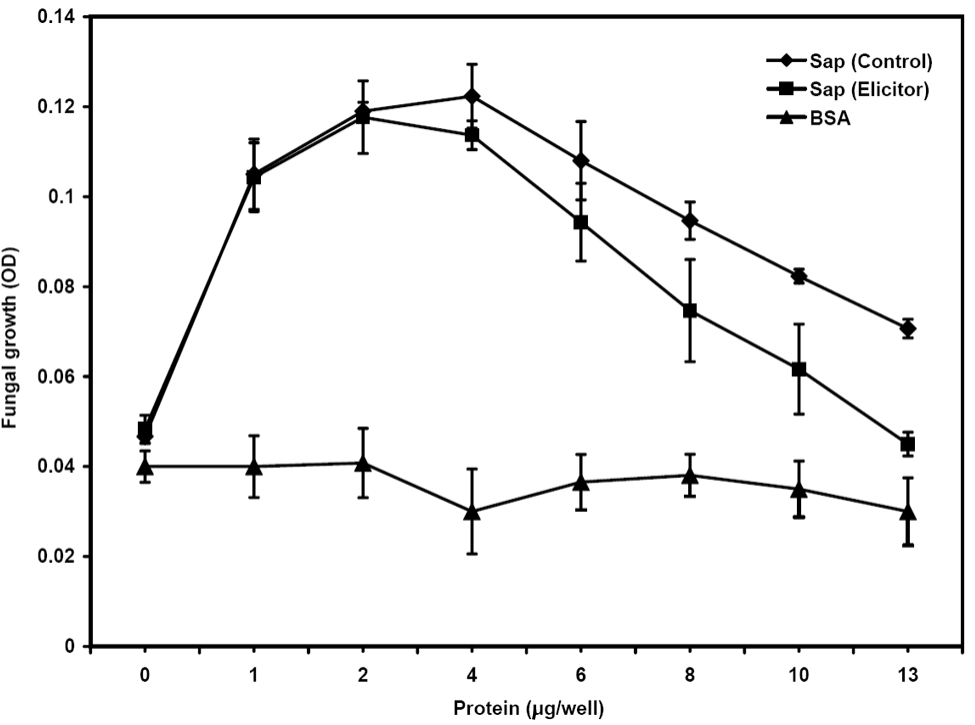
**Effect of high molecular weight xylem sap collected from control or elicitor-treated soybean plants on the growth of *N. crassa *spores as assayed by measuring OD_600_**. Addition of BSA had no effect on growth.

Corn xylem sap proteins have been shown to possess anti-fungal activity against *Neurospora crassa *[28]. We also examined the ability of soybean xylem sap to inhibit the growth of *N. crassa*. We size fractionated the xylem sap to separate small molecules and proteins less than 1 kD (LMW fraction) from larger proteins (HMW fraction) by centrifugal filtration. In order to compensate for the concentration of HMW fraction during this process, the fraction was rediluted to the same volume as LMW fraction there by restoring original sap protein concentration. We then added increasing amounts of either HMW fraction or LMW fraction of the sap to *N. crassa *spore suspension and monitored hyphae growth by microscopy and optical density measurements over a period of 24 h. When the high molecular fraction (HMW, > 1 kD) was added to the fungal spores (~1500 spores per well), we observed no inhibition of fungal growth (Figure 4); rather, we observed an increase in fungal growth with increasing amounts of sap (up to ~4 μg HMW protein per well containing ~1500 spores). This growth promotion was not observed in wells in which the same amounts of LMW fraction of xylem sap were added (data not shown). This observation suggested that proteins in the HMW fraction of soybean xylem sap might support fungal growth. However, addition of more HMW sap progressively inhibited the observed promotion of growth. Fungal growth in wells with ~13 μg of HMW proteins was not significantly different from wells with no sap proteins added (Figure 4). When we tested for promotion or inhibition of fungal growth by bovine serum albumin (BSA), we observed no significant difference in growth (Figure 4). Therefore, it is unlikely that *N. crassa *merely used xylem sap proteins as a nutrition source. It is likely that some proteins in HMW portion of the sap specifically promote fungal growth and some inhibit fungal growth. With the addition of HMW sap at levels higher than 4 μg protein per well (i.e. ~1500 spores), perhaps there was a saturation of growth promotion. However, this might increase the levels of inhibitory proteins thus causing a reduction in fungal growth. It remains to be seen what kind of proteins in the xylem sap promote fungal growth and if the sap has similar influence on the growth of fungi capable of infecting soybean plants.

We also tested if xylem sap from WGE-treated plants is capable of influencing the growth of *N. crassa*. The HMW fraction of the sap was also capable of promoting fungal growth at lower concentrations similar to that from untreated plants (Figure 4). However, at higher concentrations, HMW sap from WGE-treated plants had significantly higher inhibition of growth promotion compared to the sap from untreated plants (Figure 4). It is possible that the concentration of inhibitory proteins was higher in this sap. Nevertheless, analysis of sap composition by 2-D gel electrophoresis as mentioned above did not reveal any increase in known growth inhibitory proteins.

### Xylem sap composition in response to *B. japonicum *inoculation

Soybean plants form a symbiotic association with the nitrogen-fixing soil bacterium *B. japonicum*. We wanted to study xylem sap proteome composition in response to *B. japonicum *inoculation. It has been postulated that transmission of signals from the root to the shoot and vice versa regulate nodulation in legumes [34,38]. However, the identity of these signals are not known [42]. A recent study of soybean xylem sap at various time points starting from 2 days post inoculation did not identify any significant changes in xylem sap composition in response to *B. japonicum *[43]. We attempted to identify changes if any in the soybean xylem sap proteome at 8 h post inoculation. We chose this time point based on changes in the expression of signaling elements and flavonoid biosynthetic enzymes in soybean roots upon *B. japonicum *inoculation [53,54]. Additionally, we also wanted to compare compositional changes in the sap proteome between responses to a symbiotic and pathogenic interaction. In contrast to *P. sojae *WGE treatment, *B. japonicum *inoculation did not cause any significant increase in xylem sap protein concentration. While sap collected from mock-inoculated seedlings had approximately 7.72 μg mL^-1 ^of protein, sap from *B. japonicum*-inoculated seedlings had approximately 7.51 μg mL^-1 ^of protein.

We then examined the sap proteome by 2D gel electrophoresis. We observed increased levels of protein spots identified as xyloglucan *trans*-endoglycosyl transferase (XET, spot no. 15), malate dehydrogenase (spot no. 17), cyclophilin (spot no. 16), and a proteosome subunit (spot no. 18). We also observed decreased levels of spots identified as gamma-glutamyl hydrolase (spot no. 5), secretory peroxidase (spot no. 20) and acid phosphatase (spot no. 12) (Table 1). Of these proteins, XET was repeatedly and consistently observed to increase in response to *B. japonicum *inoculation (Figure 3C, D). XETs are involved in cell wall building and modification [55]. While it is known that cell division occurs in response to *B. japonicum *in cortical cells at ~48 h post inoculation, no such division has been known to occur at earlier time points or in xylem tissues. However, systemic induction of an XET has been observed during the formation of mycorrhizal roots in *M. truncatula *[56].

**Figure 5:**
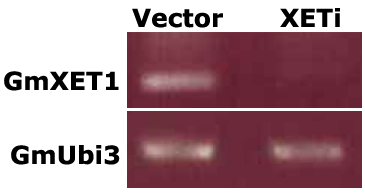
**RTPCR analysis of GmXET1 and GmUbi3 expression in transgenic soybean roots expressing a control RNAi construct (Vector) or an RNAi construct to silence GmXET1(XETi)**. PCR products were separated on an agarose gel after 18 cycles of amplification using cDNA as template.

We wanted to examine the significance of this observed increase in xylem sap XET levels during *B japonicum *colonization. We silenced the expression of this protein in composite hairy root plants via RNA interference. Transgenic roots expressing an RNAi molecule targeting GmXET1 (TA42786_3847) had reduced or no detectable expression of XET in roots (Figure 5). We then tested the effect of silencing of XET on nodule development. Silencing of XET did not significantly affect nodulation. While vector control roots expressing a control RNAi molecule targeting chloramphenicol acetyl transferase produced 7.3 ± 3.2 nodules per root, XET RNAi roots produced 8.4 ± 2.5 nodule per root. This observation suggested that GmXET1 (encoded by TA42786_3847) might not play a crucial role in nodulation under our assay conditions.

## Conclusion

We identified a number of xylem sap proteins with differential abundance during early stages of pathogenic or symbiotic microbe interactions in soybean. We also identified fungal growth promotion and inhibition activities in the soybean xylem sap. Growth inhibition activities were enhanced in sap obtained from plants challenged with pathogenic molecules. A xyloglucan *trans*-endoglycosyl transferase induced in response to rhizobium inoculation was identified. However, the function of this protein is still not clear and it might not be crucial for symbiotic nodule development.

## Methods

### Plant material and growth conditions

Approximately 200 seeds of soybean (*Glycin max *L. cv William 82) were sown into a pot (210 mm diameter X 210 mm height, Classic 600 Nursely Supplies Inc. Chambersburg, PA, USA) containing MetroMix 702 soil (The Scotts Company, Marysville, OH, USA). The seeds were germinated and the seedlings were grown in a greenhouse (26°C, 14 h photoperiod, 750 μEs). Seven-day-old young seedlings were used for elicitor and nodulation treatments.

### Rhizobium inoculation and elicitor treatment

Elicitor and *B. japonicum *treatments were done 7 days after sowing. *B. japonicum *cells were cultured and inoculated as described previously [57] except that 100 mL of the bacterial suspension (per pot) was applied to the roots. *P. sojae *WGE (a kind gift from Dr Terry Graham, Ohio State University, Columbus, OH, USA) diluted in nutrient solution (10 μg mL^-1^) was applied to the roots.

### Xylem sap collection

Sap was collected 8 h after *B. japonicum *or WGE treatment. Seedlings were de-capitated by severing the hypocotyls 2 cm above the soil surface with a sterile surgical blade. The first exudates were gently absorbed with a wet lint-free wipe (Kimwipes^®^) and discarded to avoid any potential contamination from phloem sap. Xylem sap budding from the cut surface was collected with a pipette tip (without touching the plant tissue) and pooled in a 50 mL centrifuge tube on dry ice. After the addition of EDTA-free Protease Inhibitor Cocktail (Roche Diagnostics, Penzberg, Germany; one tablet per 50 mL), the sap was stored at -80°C until further analysis. Sap was collected through eight separate experiments for each treatment. Samples from six of these experiments were used for 2D gel analyses and the rest for other assays.

### Protein purification and quantification

Frozen sap was thawed on ice, mixed well and filtered through sterile 0.2 μm cellulose acetate filters, concentrated and size-separated using a Macrosep 1 K Omega (PALL^® ^Life Sciences Centrifugal Devices, East Hills, NY, USA) centrifugal filter at 4°C. High molecular weight fractions for 2D gel electrophoresis were treated with 10% w/v trichloroaceticacid and the precipitated proteins recovered by centrifugation. Protein pellets were washed three times with 100% methanol, air-dried for 5 min, and then re-dissolved in Sequential Extraction Buffer 3 (Bio-Rad, Hercules, CA, USA) for gel electrophoresis or sterile Milli-Q water for other assays. Protein concentrations were determined by using RC-DC Protein Assay (Bio-Rad) or CB-X^® ^Protein Assay (G BioSciences, St. Louis, MO, USA) using BSA as the standard.

### 2-D gel electrophoresis

Approximately 200 μg of protein (re-suspended in 180 μL Sequential Extraction Buffer 3) was applied to an immobilized pH gradient (IPG) strip (110 mm, pH 3-10, nonlinear, Bio-Rad) and actively rehydrated. Isoelectric focusing (IEF) was performed at 20°C using a PROTEAN IEF cell (Bio-Rad) at 500 V for 1 h, 1000 V for 1 h, 2000 V for 2 h, and then at 8000 V for a total of 35 kVh. Equilibrated strips were loaded onto a gel (Criterion 8-16% gradient pre-cast 110 mm; Bio-Rad). The second dimension electrophoresis was performed at 200 V for approximately 2.5 h at 4°C. Bio-Rad broad range molecular weight markers were used to estimate molecular weight of protein spots of interest.

### Protein staining and spot intensity comparison

After electrophoresis, gels were rinsed three times with Milli-Q water, proteins were fixed in 7% acetic acid, 10% methanol for 1 h, and stained overnight with Sypro Ruby (Molecular Probes, Eugene, USA). The stained gels were washed in 7% acetic acid, 10% methanol for 1 h, rinsed with Milli-Q water and visualized using the Typhoon 9410 system (Amersham Biosciences, Piscataway, NJ, USA). In two different experiments (using Phoretix software), each with three independent 2D gels per sample, the proteomic pattern of untreated xylem sap was compared to sap from plants treated with either *P. sojae *elicitor or *B. japonicum*. In each experiment, three independent 2D gels per treatment were imaged, analyzed and compared. For each gel image, spot intensities were normalized by comparing the spot intensity to total intensity of all spots on the gel. Normalized spot intensities are averaged (three gels per treatment) and compared between treated and untreated samples. Spots with significantly different intensities (P < 0.05; Student's t-test) between control and treated samples were selected for further analyses.

### Protein identification by quadrupole TOF MS/MS

Protein spots of interest were identified using Phoretix 2D (Version 2004 Build 1440.1) and picked using Gelpix protein excision system (Genetix Ltd., Hampshire, UK). Gel slices containing the protein spots were de-stained extensively with 100% acetonitrile for 15 min and 50 mM NH_4_HCO_3_/50% acetonitrile for 15 min four times, followed by dehydration in 100% aceonitrile for 5 min. Proteins were then digested for 10 h at 37°C in 30 μL of 50 mM NH_4_HCO_3 _containing 6 μg mL^-1 ^trypsin (Promega, Madison, USA). The digested supernatant was transferred to a new tube. The gel pieces were extracted twice and the pooled digest was lyophilized and re-suspended in the aqueous buffer (10 μL of 1% formic acid/2% acetonitrile). Mini-reversed-phase column chromatography using ZipTip μ-c18 (Millipore Corporation, Billerica, MA, USA) was used for cleanup and concentration of peptides. Briefly, ZipTip was washed with 10 μL acetonitrlile, then 10 μL 60% acetonitlile/0.1% formic acid, and then 10 μL 0.1% formic acid 5 times. Ten μL sample was pipetted up and down three times to load sample and the liquid was discarded, then the ZipTip was washed with 10 μL 0.1% formic acid 4 times to remove buffers and salts. The sample was eluted with 5 μL 60% acetonitlile/0.1% formic acid. An ABI QSTAR^® ^XL (Applied Biosystems/MDS Sciex, Foster City, CA, USA) hybrid quadrupole TOF MS/MS system equipped with a nanoelectrospray source (Protana XYZ manipulator) was used for peptide sequence analysis. The nanoelectrospray was generated from a PicoTip needle (10 μm i.d., New Objectives, Wobum, MA, USA) at a voltage of 2400 V. TOF MS spectra and product ion spectra were acquired using Analyst QS software.

Each raw data file (wiff) were converted to 'peak lists' (monoisotopic and charge states calculated) and the peptide tandem mass spectra were searched against the TIGR soybean EST database (release 2 dated 09/28/2006; ) using MASCOT Daemon (version 2.2.0) search engine  with a mass tolerance of 100 ppm and one allowed trypsin miscleavage. Search parameters used the fixed cysteine carbamidomethylation and the variable methionine oxidation as modifications, and matches were judged by the number of peptide sequence tags, sequence coverage, MOWSE score, and the quality of tandem MS spectra (See Additional File 1). Positive matches were BLAST searched against entries in the Uniprot database  for updated annotation and identification of full-length sequences or homologous proteins. Presence of signal peptide-like sequences were identified based on information in the database and confirmed using SignalP analysis . Theoretical molecular masses and pI values for mature peptides were estimated using exPASy .

### Fungal growth assays

Antifungal activity of xylem sap proteins were assayed *in vitro *using 96-well microtiter plates as described previously [58]. *Neurospora crassa *spores were harvested from Vogel's medium N agar plates [59]. Fifty μl of *Neurospora *spore suspension prepared in 2× synthetic low-salt fungal medium [58] at a concentration of ~2000 spores mL^-1 ^was added to each well. Fifty μl of each protein dilution were added to each well of the microtiter plate. The fungal cultures were incubated at room temperature for 24 h and growth assayed by measuring OD_595 _values in a microtiter plate reader (Spectra MAX PLUS, Molecular Devices, Sunnyvale, CA, USA). Data was collected in at least three independent experiments.

### GmXET silencing and nodulation assays

A construct to silence GmXET1 (encoded by TA42786_3847) in soybean hairy roots was generated using Gateway *in vitro *recombination as described before for GmPR2 silencing [20]. The RNAi fragment (~265 bp) targeting GmXET1i was amplified from soybean root cDNA by PCR using the primers, GmXET1i-F 5'-TTTGTGGTGGCAGCCACAGCTGGTAGC-3' and GmXET1i-R 5'-AGTCTATTTCGTCATGAGTAGGCCCCAGA-3' and cloned in to pCR8 GW TOPO (Invitrogen, Carlsbad, CA, USA) to generate the Entry vector for use in the *in vitro *recombination reaction above [20]. Procedures for the generation of hairy root composite plants [60], nodulation assays [57] and RT-PCR analyses [57] have been previously described. Primers used for RT-PCR analyses of GmXET1 were 5'-ATGTGTGTCACGTTCATGAGGATC-3' (Forward) and 5'-CTGGCCAATCACTGGACGCCACG-3' (Reverse).

## Authors' contributions

SS and OY designed the experiments; SS, U-HC and CK performed the experiments and analyzed the data; SS and U-HC wrote the manuscript; All authors read and approved the manuscript.

## Supplementary Material

Additional File 1 **List of matching peptide sequences and related information**. The matching peptide sequences, charge state, confidence indicators and ranking used in identification of protein spots listed in the Table. Click here for file
