# Supplementary material for: Distinct changes in soybean xylem sap proteome in response to pathogenic and symbiotic microbe interactions
Source: BMC Plant Biol. 2009 Sep 21;9:119. doi: 10.1186/1471-2229-9-119 (PMC2758885; doi:10.1186/1471-2229-9-119)
Supplement: Additional File 1 — List of matching peptide sequences and related information. The matching peptide sequences, charge state, confidence indicators and ranking used in identification of protein spots listed in the Table. [file 1471-2229-9-119-S1.PDF]

Supplementary data

Mascot search results summary used for identification of protein spots. For each protein spot, the peptides used for identification are listed with additional information including peptide mass, Score, Ranking, Peptide sequence and charge state. If more than one peptide matched the same sequence, the one with the highest score was used to calculate the total MOWSE score (The scores not used in calculating MOWSE score are shown in parenthesis). Peptides shown in red are ranked high in matches. All peptides listed are from the same translational frame. This frame was verified to be the correct frame of translation *in vivo* or the longest available frame of translation (when full length cDNA is not available) based on protein BLAST searches.

Spot No. 1 Trypsin inhibitor A precursor [Glycine max (Soybean)] TA42304_3847 **Mass:** 21352 **Total score:** 147 **Peptides matched:** 5)

Query	Observed	Mr(expt)	Mr(calc)	Delta	Miss	Score	Rank	Peptide
3	407.2699	406.2626	406.2250	0.0377	0	3	8	IMK + Oxidation (M)
57	507.2303	1012.4461	1012.4072	0.0389	0	8	10	DAMDGWFR + Oxidation (M)
71	582.3103	1162.6061	1162.6346	-0.0284	0	44	1	GIGTIISSPYR
74	405.2282	1212.6627	1210.6709	1.9918	0	29	2	FIAEGHPLSLK
77	615.7653	1229.5161	1229.5200	-0.0039	0	66	1	VSDDEFNNYK

Spot No. 2 Kunitz trypsin inhibitor [Glycine soja (Wild soybean)] TA47822_3847 **Mass:** 17771 **Total score:** 237 **Peptides matched:** 8

Query	Observed	Mr(expt)	Mr(calc)	Delta	Miss	Score	Rank	Peptide
16	391.1991	780.3837	780.4381	-0.0544	0	36	1	LLTSGYK
47	596.3028	1190.5910	1190.6659	-0.0749	0	43	1	GIATIISTPYR
73	849.3864	1696.7583	1696.8420	-0.0837	0	(28)	1	SEWTVVNGQPEGPAVK
74	849.8888	1697.7630	1696.8420	0.9211	0	35	1	SEWTVVNGQPEGPAVK
75	567.2647	1698.7721	1696.8420	1.9302	0	(7)	9	SEWTVVNGQPEGPAVK
76	858.4173	1714.8201	1714.8929	-0.0729	0	(50)	1	YYVLPVIESSYGGIR
77	572.6167	1714.8283	1714.8929	-0.0646	0	54	1	YYVLPVIESSYGGIR
81	894.4004	1786.7862	1786.8525	-0.0663	0	70	1	IGSPPNAENGWFEIEK

Spot No. 3 Stem 31 kDa glycoprotein precursor [Glycine max (Soybean)] AI736946 **Mass:** 17825 **Total score:** 144 **Peptides matched:** 4

Query	Observed	Mr(expt)	Mr(calc)	Delta	Miss	Score	Rank	Peptide

9	382.2122	762.4098	762.4639	-0.0541	0	40	1	LVSLGFK
12	403.2261	804.4376	804.4857	-0.0482	0	31	1	IIFLSGR
20	555.7839	1109.5533	1109.6080	-0.0547	0	29	2	GNAPALPETLK
33	680.8169	1359.6192	1359.6571	-0.0379	0	44	1	TVNQQAYFYAR

Spot No. 4 Stem 31 kDa glycoprotein precursor [Glycine max (Soybean)] TA42145_3847 **Mass:** 30002 **Total score:** 385 **Peptides matched:** 13

Query	Observed	Mr(expt)	Mr(calc)	Delta	Miss	Score	Rank	Peptide
14	389.2216	776.4287	776.4796	-0.0509	0	39	1	LLSLGFK
16	396.2177	790.4208	790.4701	-0.0493	0	31	1	IVFLSGR
27	496.7186	991.4227	991.5008	-0.0781	0	20	1	MAVTEANLK + Oxidation (M)
28	511.7513	1021.4880	1021.5668	-0.0789	0	43	1	LAVEAHNIR
33	555.7710	1109.5275	1110.5920	-1.0645	0	26	2	GDAPALPETLK
50	481.5575	1441.6506	1441.7717	-0.1212	0	(41)	1	AGFHTWEQLILK
51	721.8607	1441.7069	1441.7717	-0.0649	0	47	1	AGFHTWEQLILK
52	734.3371	1466.6597	1467.7721	-1.1124	0	15	3	DPHLITPNALSYK
57	780.8281	1559.6416	1559.7368	-0.0951	0	40	1	TVNQQAFFYASER
71	631.9530	1892.8371	1892.9744	-0.1373	0	(48)	1	IVGIIGDQWSDLLGDHR
72	947.4535	1892.8924	1892.9744	-0.0820	0	73	1	IVGIIGDQWSDLLGDHR
90	1489.1599	2976.3053	2976.4657	-0.1603	0	57	1	EVHHNDIFIFGIDNTVLSNIPYYEK
91	745.1041	2976.3873	2976.4657	-0.0784	0	(41)	1	EVHHNDIFIFGIDNTVLSNIPYYEK

Spot No. 5 Gamma-glutamyl hydrolase precursor [Glycine max (Soybean)] TA43569_3847 Mass: 24257 Total score: 482 Peptides matched: 19

Query	Observed	Mr(expt)	Mr(calc)	Delta	Miss	Score	Rank	Peptide
12	354.1975	706.3804	706.3762	0.0042	0	24	4	GSLFQR
71	567.2542	1132.4938	1132.5076	-0.0138	0	13	6	GYDQVYLFE
74	581.2747	1160.5349	1160.6441	-0.1092	0	(41)	1	FPSDLLTQLK
75	581.3193	1160.6240	1160.6441	-0.0201	0	44	1	FPSDLLTQLK
76	581.3218	1160.6291	1160.6441	-0.0150	0	(27)	1	FPSDLLTQLK
77	581.3254	1160.6363	1160.6441	-0.0078	0	(27)	1	FPSDLLTQLK
78	581.3390	1160.6635	1160.6441	0.0194	0	(28)	1	FPSDLLTQLK
79	581.3424	1160.6702	1160.6441	0.0261	0	(28)	1	FPSDLLTQLK

80	581.3431	1160.6717	1160.6441	0.0276	0	(31)	1	FPSDLLTQLK
81	581.3438	1160.6730	1160.6441	0.0289	0	(19)	1	FPSDLLTQLK
82	581.3460	1160.6775	1160.6441	0.0334	0	(41)	1	FPSDLLTQLK
85	583.7669	1165.5192	1165.5767	-0.0575	0	43	1	NAFEWATSLK
131	737.3871	1472.7597	1472.7663	-0.0066	0	57	1	YPVTVNLWQPEK
146	524.2741	1569.8005	1569.7787	0.0219	0	(66)	1	VTQSTANFFISEAR
147	786.3900	1570.7654	1569.7787	0.9868	0	101	1	VTQSTANFFISEAR
149	534.6303	1600.8692	1600.8613	0.0079	1	43	1	KYPVTVNLWQPEK
180	620.2956	1857.8650	1857.9260	-0.0610	0	47	1	DNLIYNYKPTFGGTAGK
184	648.3149	1941.9230	1941.9319	-0.0089	1	52	1	LSDFFEILATSEDRDGK
187	529.2831	2113.1033	2113.0955	0.0078	1	61	1	VRDNLIYNYKPTFGGTAGK

Spot No. 6 CPRD14 protein – Cinnamoyl alcohol dehydrogenase [Vigna unguiculata (cowpea)] TA46458_3847 **Mass:** 34869 **Total score:** 537
Peptides matched: 19

Query	Observed	Mr(expt)	Mr(calc)	Delta	Miss	Score	Rank	Peptide
17	372.7344	743.4543	743.4541	0.0003	0	(39)	1	GTLNVLK
18	372.7399	743.4652	743.4541	0.0111	0	43	1	GTLNVLK
33	420.2023	838.3901	838.4007	-0.0105	0	(18)	2	YCLVER
34	420.2140	838.4134	838.4007	0.0127	0	19	2	YCLVER
56	502.7552	1003.4959	1003.4974	-0.0015	0	50	1	TLAEDAAWK
96	594.8060	1187.5974	1187.5968	0.0006	0	45	1	SCVNLPTLER
97	594.8112	1187.6078	1187.5968	0.0110	0	(37)	1	SCVNLPTLER
112	644.7836	1287.5527	1287.5805	-0.0278	0	30	1	ESQMWYTLSK + Oxidation (M)
118	658.8114	1315.6083	1315.7023	-0.0940	0	(30)	1	DLYPTLQLPEK
119	439.5780	1315.7121	1315.7023	0.0097	0	55	1	DLYPTLQLPEK
143	774.9383	1547.8620	1547.8446	0.0174	0	64	1	SLGIEYTPLEVSLK
158	866.5034	1730.9922	1730.9678	0.0243	0	106	1	VVLTSSVAAVAYNGKPR
159	578.3227	1731.9462	1730.9678	0.9784	0	(48)	1	VVLTSSVAAVAYNGKPR
160	578.3251	1731.9535	1730.9678	0.9857	0	(63)	1	VVLTSSVAAVAYNGKPR
161	578.3267	1731.9581	1730.9678	0.9903	0	(100)	1	VVLTSSVAAVAYNGKPR
162	434.0059	1731.9944	1730.9678	1.0265	0	(18)	1	VVLTSSVAAVAYNGKPR
171	884.4146	1766.8147	1767.8137	-0.9991	0	(31)	1	CADDNPYVPIYQVSK

172	884.9139	1767.8133	1767.8137	-0.0005	0	59	1	CADDNPYVPIYQVSK
182	629.3278	1884.9615	1884.9077	0.0537	0	66	1	DVANAHILAYENASANGR

Spot No. 7 Kunitz trypsin inhibitor [Glycine soja (Wild soybean)] TA47822_3847 **Mass:** 17999 **Total score:** 341 **Peptides matched:** 24

Query	Observed	Mr(expt)	Mr(calc)	Delta	Miss	Score	Rank	Peptide
70	596.0668	1190.1190	1190.6659	-0.5468	0	61	1	GIATIISTPYR
71	397.7327	1190.1762	1190.6659	-0.4896	0	(36)	1	GIATIISTPYR
72	596.1365	1190.2584	1190.6659	-0.4074	0	(46)	1	GIATIISTPYR
73	596.1389	1190.2633	1190.6659	-0.4026	0	(48)	1	GIATIISTPYR
74	596.1546	1190.2947	1190.6659	-0.3712	0	(48)	1	GIATIISTPYR
75	596.1587	1190.3029	1190.6659	-0.3629	0	(48)	1	GIATIISTPYR
76	596.1604	1190.3063	1190.6659	-0.3596	0	(50)	1	GIATIISTPYR
77	596.1613	1190.3081	1190.6659	-0.3578	0	(48)	1	GIATIISTPYR
78	596.1622	1190.3099	1190.6659	-0.3560	0	(48)	1	GIATIISTPYR
79	596.1623	1190.3101	1190.6659	-0.3558	0	(50)	1	GIATIISTPYR
80	596.1636	1190.3126	1190.6659	-0.3533	0	(50)	1	GIATIISTPYR
103	796.1454	1590.2763	1591.7552	-1.4788	0	(54)	1	CPLTVVQSADPYDK
104	796.1497	1590.2848	1591.7552	-1.4704	0	(64)	1	CPLTVVQSADPYDK
105	796.1499	1590.2853	1591.7552	-1.4699	0	(75)	1	CPLTVVQSADPYDK
106	531.4096	1591.2068	1591.7552	-0.5483	0	(53)	1	CPLTVVQSADPYDK
107	796.6354	1591.2562	1591.7552	-0.4990	0	(79)	1	CPLTVVQSADPYDK
108	796.6578	1591.3011	1591.7552	-0.4541	0	84	1	CPLTVVQSADPYDK
109	796.6699	1591.3252	1591.7552	-0.4300	0	(72)	1	CPLTVVQSADPYDK
126	849.1635	1696.3125	1696.8420	-0.5295	0	(16)	1	SEWTVVNGQPEGPAVK
127	567.0928	1698.2566	1696.8420	1.4146	0	24	1	SEWTVVNGQPEGPAVK
135	572.4607	1714.3602	1714.8929	-0.5327	0	(86)	1	YYVLPVIESSYGGIR
136	858.2269	1714.4392	1714.8929	-0.4537	0	95	1	YYVLPVIESSYGGIR
160	585.8410	1754.5013	1755.0406	-0.5393	1	25	1	GIATIISTPYRVPVIR
171	626.4558	1876.3455	1876.8989	-0.5534	1	52	1	ERCPLTVVQSADPYDK

Spot No. 8 Trypsin inhibitor A precursor [Glycine max (Soybean)] TA42304_3847 **Mass:** 18003 **Total score:** 317 **Peptides matched:** 21

Query	Observed	Mr(expt)	Mr(calc)	Delta	Miss	Score	Rank	Peptide
70	596.0668	1190.1190	1190.6659	-0.5468	0	61	1	GIATIISTPYR
71	397.7327	1190.1762	1190.6659	-0.4896	0	(36)	1	GIATIISTPYR
72	596.1365	1190.2584	1190.6659	-0.4074	0	(46)	1	GIATIISTPYR
73	596.1389	1190.2633	1190.6659	-0.4026	0	(48)	1	GIATIISTPYR
74	596.1546	1190.2947	1190.6659	-0.3712	0	(48)	1	GIATIISTPYR
75	596.1587	1190.3029	1190.6659	-0.3629	0	(48)	1	GIATIISTPYR
76	596.1604	1190.3063	1190.6659	-0.3596	0	(50)	1	GIATIISTPYR
77	596.1613	1190.3081	1190.6659	-0.3578	0	(48)	1	GIATIISTPYR
78	596.1622	1190.3099	1190.6659	-0.3560	0	(48)	1	GIATIISTPYR
79	596.1623	1190.3101	1190.6659	-0.3558	0	(50)	1	GIATIISTPYR
80	596.1636	1190.3126	1190.6659	-0.3533	0	(50)	1	GIATIISTPYR
106	531.4096	1591.2068	1592.7392	-1.5323	0	(46)	1	CPLTVVQSADPYDK
107	796.6354	1591.2562	1592.7392	-1.4830	0	(72)	1	CPLTVVQSADPYDK
108	796.6578	1591.3011	1592.7392	-1.4381	0	77	1	CPLTVVQSADPYDK
109	796.6699	1591.3252	1592.7392	-1.4140	0	(61)	1	CPLTVVQSADPYDK
126	849.1635	1696.3125	1696.8420	-0.5295	0	(16)	1	SEWTVVNGQPEGPAVK
127	567.0928	1698.2566	1696.8420	1.4146	0	24	1	SEWTVVNGQPEGPAVK
135	572.4607	1714.3602	1714.8929	-0.5327	0	(86)	1	YYVLPVIESSYGGIR
136	858.2269	1714.4392	1714.8929	-0.4537	0	95	1	YYVLPVIESSYGGIR
160	585.8410	1754.5013	1755.0406	-0.5393	1	25	1	GIATIISTPYRVPVIR
171	626.4558	1876.3455	1877.8829	-1.5374	1	34	1	ERCPLTVVQSADPYDK

Spot No. 9 Trypsin inhibitor A precursor [Glycine max (Soybean)] TA42304_3847 **Mass:** 22623 **Total score:** 247 **Peptides matched:** 40

Query	Observed	Mr(expt)	Mr(calc)	Delta	Miss	Score	Rank	Peptide
86	529.7495	1057.4845	1058.5542	-1.0697	0	(41)	1	CPLTVVQSR
87	529.7500	1057.4854	1058.5542	-1.0688	0	(41)	1	CPLTVVQSR
88	530.2521	1058.4897	1058.5542	-0.0646	0	(35)	1	CPLTVVQSR
89	530.2551	1058.4956	1058.5542	-0.0586	0	(24)	1	CPLTVVQSR
90	530.2583	1058.5021	1058.5542	-0.0521	0	43	1	CPLTVVQSR
91	530.2644	1058.5143	1058.5542	-0.0399	0	(41)	1	CPLTVVQSR

92	530.2649	1058.5152	1058.5542	-0.0390	0	(34)	1	CPLTVVQSR
93	530.2707	1058.5268	1058.5542	-0.0274	0	(38)	1	CPLTVVQSR
94	530.2793	1058.5441	1058.5542	-0.0102	0	(38)	1	CPLTVVQSR
95	530.2808	1058.5470	1058.5542	-0.0072	0	(35)	1	CPLTVVQSR
96	530.2820	1058.5494	1058.5542	-0.0048	0	(42)	1	CPLTVVQSR
97	530.2899	1058.5652	1058.5542	0.0110	0	(33)	1	CPLTVVQSR
126	582.2938	1162.5730	1162.6346	-0.0615	0	(47)	1	GIGTIISSPYR
127	582.2988	1162.5830	1162.6346	-0.0515	0	(49)	1	GIGTIISSPYR
128	582.3057	1162.5968	1162.6346	-0.0377	0	(48)	1	GIGTIISSPYR
129	582.3064	1162.5982	1162.6346	-0.0364	0	(48)	1	GIGTIISSPYR
130	582.3112	1162.6078	1162.6346	-0.0268	0	(54)	1	GIGTIISSPYR
131	582.3168	1162.6191	1162.6346	-0.0154	0	(51)	1	GIGTIISSPYR
132	582.3212	1162.6278	1162.6346	-0.0068	0	58	1	GIGTIISSPYR
133	582.3215	1162.6285	1162.6346	-0.0061	0	(48)	1	GIGTIISSPYR
134	582.3216	1162.6287	1162.6346	-0.0059	0	(44)	1	GIGTIISSPYR
151	404.5148	1210.5226	1210.6709	-0.1484	0	(35)	1	FIAEGHPLSLK
152	404.5402	1210.5988	1210.6709	-0.0721	0	(33)	1	FIAEGHPLSLK
153	404.5435	1210.6088	1210.6709	-0.0622	0	(34)	1	FIAEGHPLSLK
154	606.3128	1210.6111	1210.6709	-0.0598	0	61	1	FIAEGHPLSLK
155	404.5507	1210.6302	1210.6709	-0.0407	0	(42)	1	FIAEGHPLSLK
156	606.3268	1210.6391	1210.6709	-0.0318	0	(28)	1	FIAEGHPLSLK
157	606.3284	1210.6422	1210.6709	-0.0287	0	(37)	1	FIAEGHPLSLK
158	404.5572	1210.6499	1210.6709	-0.0210	0	(39)	1	FIAEGHPLSLK
159	404.5645	1210.6716	1210.6709	0.0006	0	(34)	1	FIAEGHPLSLK
160	404.5654	1210.6743	1210.6709	0.0034	0	(38)	1	FIAEGHPLSLK
161	404.5661	1210.6764	1210.6709	0.0054	0	(37)	1	FIAEGHPLSLK
162	404.5669	1210.6789	1210.6709	0.0080	0	(34)	1	FIAEGHPLSLK
163	404.5671	1210.6795	1210.6709	0.0085	0	(33)	1	FIAEGHPLSLK
164	404.5699	1210.6879	1210.6709	0.0170	0	(31)	1	FIAEGHPLSLK
165	404.8861	1211.6365	1210.6709	0.9655	0	(41)	1	FIAEGHPLSLK
166	405.2159	1212.6258	1210.6709	1.9548	0	(44)	1	FIAEGHPLSLK
210	588.2982	1761.8727	1761.9260	-0.0533	1	75	1	NELDKGIGTIISSPYR
217	624.2855	1869.8348	1870.9319	-1.0971	1	11	4	ATPSGNERCPLTVVQSR

219	624.9737	1871.8992	1870.9319	0.9673	1	(5)	8	ATPSGNERCPLTVVQSR

Spot no. 10 Stem 31 kDa glycoprotein precursor [Glycine max (Soybean)] TA41989_3847 **Mass:** 30189 **Total score:** 705 **Peptides matched:** 83

Query	Observed	Mr(expt)	Mr(calc)	Delta	Miss	Score	Rank	Peptide
10	340.0580	678.1015	678.2908	-0.1893	0	20	1	CASWR
15	382.0717	762.1288	762.4639	-0.3351	0	(38)	1	LVSLGFK
16	382.0718	762.1290	762.4639	-0.3349	0	(38)	1	LVSLGFK
17	382.0719	762.1292	762.4639	-0.3347	0	(38)	1	LVSLGFK
18	382.0727	762.1309	762.4639	-0.3331	0	(39)	1	LVSLGFK
19	382.0728	762.1311	762.4639	-0.3328	0	(33)	1	LVSLGFK
20	382.0764	762.1383	762.4639	-0.3256	0	40	1	LVSLGFK
21	382.0831	762.1517	762.4639	-0.3122	0	(36)	1	LVSLGFK
28	395.0447	788.0749	788.3816	-0.3068	0	(20)	1	HGYGVEK
29	395.0464	788.0783	788.3816	-0.3034	0	(22)	2	HGYGVEK
30	395.0468	788.0790	788.3816	-0.3026	0	(19)	1	HGYGVEK
31	395.0469	788.0792	788.3816	-0.3025	0	(16)	1	HGYGVEK
32	395.0488	788.0830	788.3816	-0.2987	0	(22)	1	HGYGVEK
33	395.0496	788.0846	788.3816	-0.2970	0	(23)	1	HGYGVEK
34	395.0507	788.0869	788.3816	-0.2948	0	(33)	1	HGYGVEK
35	395.0517	788.0889	788.3816	-0.2928	0	(29)	1	HGYGVEK
36	395.0518	788.0890	788.3816	-0.2927	0	(19)	2	HGYGVEK
37	395.0525	788.0904	788.3816	-0.2912	0	(25)	1	HGYGVEK
38	395.0581	788.1016	788.3816	-0.2800	0	45	1	HGYGVEK
39	395.0609	788.1072	788.3816	-0.2744	0	(43)	1	HGYGVEK
42	403.0567	804.0988	804.4857	-0.3869	0	(32)	2	IIFLSGR
43	403.0754	804.1362	804.4857	-0.3495	0	(33)	5	IIFLSGR
44	403.0776	804.1406	804.4857	-0.3451	0	(33)	2	IIFLSGR
45	403.0805	804.1464	804.4857	-0.3393	0	(33)	2	IIFLSGR
46	403.0828	804.1510	804.4857	-0.3347	0	(33)	2	IIFLSGR
47	403.0898	804.1650	804.4857	-0.3207	0	33	2	IIFLSGR
48	404.0837	806.1529	804.4857	1.6672	0	(33)	1	IIFLSGR
54	419.0785	836.1425	836.4392	-0.2967	0	(16)	1	DLEVHPK

55	419.0794	836.1443	836.4392	-0.2949	0	(33)	1	DLEVHPK
56	419.0795	836.1445	836.4392	-0.2947	0	(29)	1	DLEVHPK
57	419.0804	836.1462	836.4392	-0.2930	0	(19)	1	DLEVHPK
58	419.0806	836.1466	836.4392	-0.2925	0	(30)	1	DLEVHPK
59	419.0814	836.1483	836.4392	-0.2909	0	35	1	DLEVHPK
60	419.0831	836.1516	836.4392	-0.2876	0	(30)	1	DLEVHPK
61	419.0861	836.1577	836.4392	-0.2815	0	(33)	1	DLEVHPK
87	487.0750	972.1354	972.5240	-0.3886	0	(30)	1	QAVTEANLK
88	487.0791	972.1436	972.5240	-0.3804	0	(33)	1	QAVTEANLK
89	487.0828	972.1510	972.5240	-0.3730	0	(40)	1	QAVTEANLK
90	487.0913	972.1681	972.5240	-0.3559	0	(32)	1	QAVTEANLK
91	487.0918	972.1691	972.5240	-0.3549	0	(45)	1	QAVTEANLK
92	487.0920	972.1695	972.5240	-0.3544	0	(41)	1	QAVTEANLK
93	487.0932	972.1718	972.5240	-0.3522	0	(46)	1	QAVTEANLK
94	487.1032	972.1918	972.5240	-0.3321	0	(29)	1	QAVTEANLK
95	487.1039	972.1932	972.5240	-0.3307	0	(52)	1	QAVTEANLK
96	487.1047	972.1949	972.5240	-0.3291	0	(41)	1	QAVTEANLK
97	487.1132	972.2119	972.5240	-0.3120	0	(51)	1	QAVTEANLK
98	487.1179	972.2213	972.5240	-0.3027	0	(48)	1	QAVTEANLK
99	487.1189	972.2233	972.5240	-0.3007	0	57	1	QAVTEANLK
100	487.1191	972.2236	972.5240	-0.3003	0	(49)	1	QAVTEANLK
101	487.1199	972.2252	972.5240	-0.2988	0	(51)	1	QAVTEANLK
102	487.1204	972.2262	972.5240	-0.2978	0	(48)	1	QAVTEANLK
103	331.0311	990.0714	990.4559	-0.3845	0	17	10	AGYHTWEK
116	555.6023	1109.1901	1109.6080	-0.4179	0	(29)	1	GNAPALPETLK
117	555.6084	1109.2023	1109.6080	-0.4057	0	(31)	1	GNAPALPETLK
118	555.6156	1109.2167	1109.6080	-0.3913	0	(34)	2	GNAPALPETLK
119	555.6157	1109.2168	1109.6080	-0.3912	0	(38)	1	GNAPALPETLK
120	555.6161	1109.2177	1109.6080	-0.3903	0	(31)	1	GNAPALPETLK
121	555.6170	1109.2194	1109.6080	-0.3886	0	42	1	GNAPALPETLK
122	555.6179	1109.2212	1109.6080	-0.3868	0	(38)	1	GNAPALPETLK
123	555.6200	1109.2254	1109.6080	-0.3826	0	(41)	1	GNAPALPETLK
166	428.0703	1281.1892	1281.7080	-0.5188	1	67	1	NYNKLVSLGFK

177	454.0472	1359.1197	1359.6571	-0.5374	0	(33)	1	TVNQQAYFYAR
178	680.5777	1359.1409	1359.6571	-0.5162	0	(46)	1	TVNQQAYFYAR
179	680.6188	1359.2231	1359.6571	-0.4340	0	(45)	1	TVNQQAYFYAR
180	680.6228	1359.2311	1359.6571	-0.4259	0	(46)	1	TVNQQAYFYAR
181	680.6236	1359.2326	1359.6571	-0.4245	0	(39)	1	TVNQQAYFYAR
182	680.6246	1359.2346	1359.6571	-0.4225	0	(46)	1	TVNQQAYFYAR
183	680.6261	1359.2377	1359.6571	-0.4194	0	(46)	1	TVNQQAYFYAR
184	680.6262	1359.2378	1359.6571	-0.4193	0	(42)	1	TVNQQAYFYAR
185	680.6283	1359.2421	1359.6571	-0.4150	0	47	1	TVNQQAYFYAR
199	506.7586	1517.2539	1517.6998	-0.4459	0	(44)	1	DPQDPSTPNAVSYK
200	759.6408	1517.2671	1517.6998	-0.4327	0	78	1	DPQDPSTPNAVSYK
201	759.6466	1517.2787	1517.6998	-0.4211	0	(74)	1	DPQDPSTPNAVSYK
246	497.0660	1984.2350	1985.0469	-0.8119	1	(45)	1	LILKDPQDPSTPNAVSYK
247	993.1850	1984.3554	1985.0469	-0.6915	1	104	1	LILKDPQDPSTPNAVSYK
248	662.4601	1984.3584	1985.0469	-0.6885	1	(67)	1	LILKDPQDPSTPNAVSYK
249	662.4787	1984.4142	1985.0469	-0.6327	1	(59)	1	LILKDPQDPSTPNAVSYK
250	662.4800	1984.4181	1985.0469	-0.6288	1	(64)	1	LILKDPQDPSTPNAVSYK
251	662.4816	1984.4230	1985.0469	-0.6239	1	(58)	1	LILKDPQDPSTPNAVSYK
252	662.4820	1984.4241	1985.0469	-0.6228	1	(69)	1	LILKDPQDPSTPNAVSYK
280	766.4970	2296.4690	2297.1552	-0.6861	0	77	1	QGYNIVGIIGDQWSDLLGGHR
281	575.1330	2296.5029	2297.1552	-0.6523	0	(65)	1	QGYNIVGIIGDQWSDLLGGHR
315	868.4555	2602.3448	2603.2576	-0.9129	0	47	1	LAVEAHNIFGFETIPEECVEATK

Spot No. 11 Stem 31 kDa glycoprotein precursor [Glycine max (Soybean)] TA41989_3847 **Mass:** 19893 **Total score:** 188 **Peptides matched:** 4

Query	Observed	Mr(expt)	Mr(calc)	Delta	Miss	Score	Rank	Peptide
19	403.0989	804.1833	804.4857	-0.3024	0	37	4	IIFLSGR
50	555.1519	1108.2893	1109.6080	-1.3187	0	12	1	GNAPALPETLK
62	662.4856	1984.4349	1985.0469	-0.6120	1	92	1	LILKDPQDPSTPNAVSYK
66	868.8777	2603.6112	2603.2576	0.3536	0	48	1	LAVEAHNIFGFETIPEECVEATK

Spot No. 12 Acid phosphatase precursor [Phaseolus vulgaris (Kidney bean) (French bean)] TA42402_3847 **Mass:** 21169 **Total score:** 392 **Peptides matched:** 15

Query	Observed	Mr(expt)	Mr(calc)	Delta	Miss	Score	Rank	Peptide
12	366.5529	731.0913	731.3854	-0.2941	0	28	1	YPIPDK
13	366.5540	731.0934	731.3854	-0.2920	0	(24)	7	YPIPDK
81	393.7117	1178.1134	1178.6084	-0.4950	0	(38)	1	STSPLWYAVR
82	590.0997	1178.1849	1178.6084	-0.4235	0	(40)	1	STSPLWYAVR
83	590.1018	1178.1890	1178.6084	-0.4193	0	50	1	STSPLWYAVR
84	591.0656	1180.1167	1178.6084	1.5083	0	(26)	1	STSPLWYAVR
98	606.1184	1210.2222	1210.6386	-0.4164	0	(9)	8	YTPQYIWLK
99	606.1373	1210.2601	1210.6386	-0.3785	0	27	1	YTPQYIWLK
110	416.0618	1245.1636	1245.6043	-0.4407	0	38	1	THAIYHWYR
121	686.6058	1371.1970	1371.6418	-0.4449	0	69	1	YSNIDYNITGGR
126	717.1294	1432.2443	1432.7026	-0.4583	0	41	1	SVFETWFVQYK
142	403.8060	1611.1947	1611.8157	-0.6210	0	63	1	VDVIFAGHVHAYER
143	538.0839	1611.2298	1611.8157	-0.5859	0	(58)	1	VDVIFAGHVHAYER
185	469.3521	1873.3792	1874.0413	-0.6622	1	(55)	1	RASAHIIVLSSYSPFVK
186	625.5066	1873.4981	1874.0413	-0.5432	1	75	1	RASAHIIVLSSYSPFVK

Spot No. 13 Stem 31 kDa glycoprotein precursor [Glycine max (Soybean)] TA42145_3847 **Mass:** 32705 **Total score:** 1042 **Peptides matched:** 107

Query	Observed	Mr(expt)	Mr(calc)	Delta	Miss	Score	Rank	Peptide
28	389.0307	776.0469	776.4796	-0.4326	0	(41)	1	LLSLGFK
29	389.0649	776.1153	776.4796	-0.3643	0	(46)	1	LLSLGFK
30	389.0661	776.1176	776.4796	-0.3619	0	46	1	LLSLGFK
31	389.0756	776.1366	776.4796	-0.3430	0	(44)	1	LLSLGFK
32	396.0517	790.0889	790.4701	-0.3812	0	(32)	1	IVFLSGR
33	396.0554	790.0962	790.4701	-0.3739	0	(33)	1	IVFLSGR
34	396.0633	790.1121	790.4701	-0.3580	0	(33)	1	IVFLSGR
35	396.0712	790.1278	790.4701	-0.3423	0	36	1	IVFLSGR
36	396.0720	790.1294	790.4701	-0.3407	0	(36)	1	IVFLSGR
88	488.5643	975.1140	975.5059	-0.3919	0	(15)	3	MAVTEANLK
89	488.5675	975.1204	975.5059	-0.3855	0	(18)	3	MAVTEANLK

90	488.5683	975.1220	975.5059	-0.3839	0	(12)	8	MAVTEANLK
91	488.5716	975.1286	975.5059	-0.3773	0	59	1	MAVTEANLK
92	488.5743	975.1340	975.5059	-0.3719	0	(47)	1	MAVTEANLK
93	488.5762	975.1379	975.5059	-0.3680	0	(47)	1	MAVTEANLK
94	488.5846	975.1547	975.5059	-0.3511	0	(40)	1	MAVTEANLK
95	496.5503	991.0859	991.5008	-0.4148	0	(15)	4	MAVTEANLK + Oxidation (M)
96	496.5662	991.1179	991.5008	-0.3829	0	(28)	1	MAVTEANLK + Oxidation (M)
97	496.5702	991.1258	991.5008	-0.3750	0	(35)	1	MAVTEANLK + Oxidation (M)
98	496.5731	991.1316	991.5008	-0.3691	0	(31)	1	MAVTEANLK + Oxidation (M)
99	496.5758	991.1370	991.5008	-0.3638	0	(38)	1	MAVTEANLK + Oxidation (M)
100	496.5787	991.1429	991.5008	-0.3579	0	(37)	1	MAVTEANLK + Oxidation (M)
101	496.5848	991.1551	991.5008	-0.3457	0	(34)	1	MAVTEANLK + Oxidation (M)
102	496.5866	991.1587	991.5008	-0.3421	0	(43)	1	MAVTEANLK + Oxidation (M)
103	496.5868	991.1591	991.5008	-0.3417	0	(40)	1	MAVTEANLK + Oxidation (M)
104	496.5876	991.1606	991.5008	-0.3402	0	(41)	1	MAVTEANLK + Oxidation (M)
105	496.5890	991.1634	991.5008	-0.3374	0	(40)	1	MAVTEANLK + Oxidation (M)
106	496.5893	991.1641	991.5008	-0.3367	0	(40)	1	MAVTEANLK + Oxidation (M)
109	341.3721	1021.0943	1021.5668	-0.4725	0	(52)	1	LAVEAHNIR
110	341.3727	1021.0961	1021.5668	-0.4707	0	(47)	3	LAVEAHNIR
111	341.3731	1021.0973	1021.5668	-0.4695	0	(47)	2	LAVEAHNIR
112	341.3731	1021.0976	1021.5668	-0.4693	0	(33)	2	LAVEAHNIR
113	341.3732	1021.0978	1021.5668	-0.4690	0	(43)	1	LAVEAHNIR
114	341.3746	1021.1020	1021.5668	-0.4648	0	(37)	3	LAVEAHNIR
115	341.3746	1021.1021	1021.5668	-0.4647	0	(49)	1	LAVEAHNIR
116	341.3748	1021.1027	1021.5668	-0.4642	0	(44)	1	LAVEAHNIR
117	341.3750	1021.1030	1021.5668	-0.4638	0	(47)	2	LAVEAHNIR
118	341.3763	1021.1071	1021.5668	-0.4598	0	(48)	1	LAVEAHNIR
119	341.3815	1021.1227	1021.5668	-0.4441	0	(43)	3	LAVEAHNIR
120	341.3831	1021.1276	1021.5668	-0.4392	0	(50)	3	LAVEAHNIR
121	511.5996	1021.1847	1021.5668	-0.3821	0	(59)	1	LAVEAHNIR
122	511.6118	1021.2091	1021.5668	-0.3578	0	(50)	3	LAVEAHNIR
123	511.6166	1021.2187	1021.5668	-0.3481	0	(51)	1	LAVEAHNIR
124	511.6167	1021.2188	1021.5668	-0.3480	0	(52)	2	LAVEAHNIR

125	511.6192	1021.2238	1021.5668	-0.3430	0	(55)	2	LAVEAHNIR
126	511.6211	1021.2276	1021.5668	-0.3392	0	61	1	LAVEAHNIR
127	511.6249	1021.2353	1021.5668	-0.3316	0	(59)	2	LAVEAHNIR
146	552.6030	1103.1915	1103.6008	-0.4094	1	25	1	MAVTEANLKK
148	555.6050	1109.1954	1110.5920	-1.3966	0	(38)	1	GDAPALPETLK
149	556.0854	1110.1563	1110.5920	-0.4357	0	(36)	2	GDAPALPETLK
150	556.0969	1110.1792	1110.5920	-0.4128	0	(37)	1	GDAPALPETLK
151	556.1019	1110.1893	1110.5920	-0.4028	0	(37)	1	GDAPALPETLK
152	556.1077	1110.2009	1110.5920	-0.3911	0	47	1	GDAPALPETLK
156	570.0470	1138.0795	1138.5369	-0.4573	0	(27)	5	LPNPMYYIE
158	570.0632	1138.1119	1138.5369	-0.4250	0	(26)	7	LPNPMYYIE
159	570.0656	1138.1167	1138.5369	-0.4202	0	(26)	2	LPNPMYYIE
160	571.0535	1140.0924	1140.5199	-0.4275	0	23	1	DYINGEQFR
165	578.0591	1154.1036	1154.5318	-0.4282	0	27	8	LPNPMYYIE + Oxidation (M)
184	432.7207	1295.1402	1295.7237	-0.5835	1	44	1	NYNKLLSLGFK
185	651.5934	1301.1723	1301.6173	-0.4450	0	40	1	TIPEECVEPTK
186	651.5968	1301.1790	1301.6173	-0.4383	0	(36)	2	TIPEECVEPTK
187	651.5986	1301.1827	1301.6173	-0.4346	0	(37)	3	TIPEECVEPTK
188	651.5989	1301.1832	1301.6173	-0.4341	0	(34)	3	TIPEECVEPTK
207	481.3903	1441.1489	1441.7717	-0.6228	0	(41)	1	AGFHTWEQLILK
208	721.5931	1441.1716	1441.7717	-0.6001	0	62	1	AGFHTWEQLILK
209	481.4194	1441.2362	1441.7717	-0.5355	0	(41)	1	AGFHTWEQLILK
210	481.4195	1441.2367	1441.7717	-0.5350	0	(42)	1	AGFHTWEQLILK
211	481.4200	1441.2383	1441.7717	-0.5335	0	(40)	1	AGFHTWEQLILK
224	490.0521	1467.1345	1467.7721	-0.6377	0	53	1	DPHLITPNALSYK
225	490.0742	1467.2006	1467.7721	-0.5715	0	(30)	1	DPHLITPNALSYK
226	734.6177	1467.2209	1467.7721	-0.5513	0	(52)	1	DPHLITPNALSYK
227	490.0833	1467.2280	1467.7721	-0.5441	0	(41)	1	DPHLITPNALSYK
228	490.0904	1467.2493	1467.7721	-0.5228	0	(33)	1	DPHLITPNALSYK
229	490.4061	1468.1964	1467.7721	0.4243	0	(26)	1	DPHLITPNALSYK
246	504.4283	1510.2631	1510.7701	-0.5070	1	40	1	YLDKMAVTEANLK + Oxidation (M)
249	766.1051	1530.1957	1530.7428	-0.5471	1	30	1	TFKLPNPMYYIE + Oxidation (M)
254	780.5245	1559.0344	1559.7368	-0.7024	0	(42)	2	TVNQQAFFYASER

255	520.6949	1559.0630	1559.7368	-0.6738	0	(41)	1	TVNQQAFFYASER
256	520.7243	1559.1511	1559.7368	-0.5857	0	(34)	2	TVNQQAFFYASER
257	520.7268	1559.1587	1559.7368	-0.5781	0	(48)	1	TVNQQAFFYASER
258	520.7316	1559.1728	1559.7368	-0.5640	0	(31)	2	TVNQQAFFYASER
259	520.7358	1559.1854	1559.7368	-0.5514	0	(38)	2	TVNQQAFFYASER
260	520.7370	1559.1890	1559.7368	-0.5478	0	(38)	1	TVNQQAFFYASER
261	780.6026	1559.1906	1559.7368	-0.5462	0	(44)	1	TVNQQAFFYASER
262	780.6137	1559.2128	1559.7368	-0.5240	0	(44)	2	TVNQQAFFYASER
263	780.6231	1559.2316	1559.7368	-0.5052	0	(41)	2	TVNQQAFFYASER
264	780.6246	1559.2346	1559.7368	-0.5022	0	(41)	2	TVNQQAFFYASER
265	781.6187	1561.2228	1559.7368	1.4860	0	50	1	TVNQQAFFYASER
266	524.0847	1569.2324	1569.8667	-0.6343	1	(16)	2	KAGFHTWEQLILK
267	785.6489	1569.2833	1569.8667	-0.5834	1	50	1	KAGFHTWEQLILK
303	631.6805	1892.0198	1892.9744	-0.9546	0	(117)	1	IVGIIGDQWSDLLGDHR
304	474.0589	1892.2065	1892.9744	-0.7679	0	(60)	1	IVGIIGDQWSDLLGDHR
305	947.1661	1892.3176	1892.9744	-0.6568	0	151	1	IVGIIGDQWSDLLGDHR
306	631.7935	1892.3588	1892.9744	-0.6156	0	(99)	1	IVGIIGDQWSDLLGDHR
307	631.8029	1892.3868	1892.9744	-0.5876	0	(86)	1	IVGIIGDQWSDLLGDHR
308	631.8032	1892.3876	1892.9744	-0.5868	0	(108)	1	IVGIIGDQWSDLLGDHR
309	631.8159	1892.4259	1892.9744	-0.5485	0	(86)	1	IVGIIGDQWSDLLGDHR
338	581.2924	2321.1407	2322.1716	-1.0309	1	43	2	IVGIIGDQWSDLLGDHRGESR
339	774.7707	2321.2903	2322.1716	-0.8812	1	(32)	1	IVGIIGDQWSDLLGDHRGESR
356	1212.6941	2423.3736	2424.1267	-0.7531	1	(74)	2	TIPEECVEPTKDYINGEQFR
357	607.0592	2424.2077	2424.1267	0.0810	1	(63)	1	TIPEECVEPTKDYINGEQFR
358	809.0956	2424.2651	2424.1267	0.1384	1	99	2	TIPEECVEPTKDYINGEQFR
359	809.0998	2424.2776	2424.1267	0.1509	1	(74)	1	TIPEECVEPTKDYINGEQFR
360	809.1289	2424.3648	2424.1267	0.2381	1	(61)	2	TIPEECVEPTKDYINGEQFR
361	809.1412	2424.4017	2424.1267	0.2750	1	(66)	1	TIPEECVEPTKDYINGEQFR
362	809.1416	2424.4029	2424.1267	0.2763	1	(91)	1	TIPEECVEPTKDYINGEQFR
368	723.6476	2890.5614	2891.5333	-0.9719	1	65	1	AGFHTWEQLILKDPHLITPNALSYK

Spot No. 14 Stem 31 kDa glycoprotein precursor [Glycine max (Soybean)] TA42145_3847 **Mass:** 20215 **Total score:** 311 **Peptides matched:** 13

Query	Observed	Mr(expt)	Mr(calc)	Delta	Miss	Score	Rank	Peptide
32	389.0696	776.1247	776.4796	-0.3548	0	(38)	1	LLSLGFK
33	389.0719	776.1292	776.4796	-0.3504	0	(40)	1	LLSLGFK
34	389.0809	776.1473	776.4796	-0.3323	0	40	1	LLSLGFK
35	389.0835	776.1525	776.4796	-0.3271	0	(40)	1	LLSLGFK
36	389.0842	776.1538	776.4796	-0.3258	0	(38)	1	LLSLGFK
37	389.0848	776.1551	776.4796	-0.3245	0	(32)	1	LLSLGFK
39	396.0824	790.1503	790.4701	-0.3197	0	36	1	IVFLSGR
205	481.4182	1441.2329	1441.7717	-0.5389	0	40	1	AGFHTWEQLILK
213	520.7320	1559.1742	1558.7779	0.3963	0	(39)	2	TVIQQAFFYASER
214	780.5990	1559.1834	1558.7779	0.4055	0	(17)	3	TVIQQAFFYASER
215	780.6176	1559.2207	1558.7779	0.4428	0	53	1	TVIQQAFFYASER
248	631.8146	1892.4220	1892.9744	-0.5524	0	99	1	IVGIIGDQWSDLLGDHR
254	581.3439	2321.3465	2322.1716	-0.8251	1	42	1	IVGIIGDQWSDLLGDHRGESR

Spot No. 15 Xyloglucan endotransglycosylase [Malus domestica (Apple) (Malus sylvestris)] TA43786_3847 **Mass:** 16689 **Total score:** 205 **Peptides matched:** 7

Query	Observed	Mr(expt)	Mr(calc)	Delta	Miss	Score	Rank	Peptide
26	456.73	911.45	911.45	0.00	0	23	1	APFTASYR
39	658.32	1314.62	1314.63	-0.01	0	22	2	FPQGLPPECSIA
42	736.38	1470.74	1470.73	0.01	1	35	1	RFPQGLPPECSIA
50	547.30	1638.87	1640.70	-1.82	0	(22)	5	NYMIYNYCTDIR + Oxidation (M)
51	821.38	1640.75	1640.70	0.05	0	44	1	NYMIYNYCTDIR + Oxidation (M)
72	710.33	2127.98	2127.97	0.01	0	(41)	1	NFNSQTSSSTGQSLDATGQAK
73	1065.03	2128.04	2127.97	0.07	0	80	1	NFNSQTSSSTGQSLDATGQAK

Spot No. 16 Cyclophilin [Kandelia candel] TA43384_3847 **Mass:** 19392 **Total score:** 89 **Peptides matched:** 4

Query	Observed	Mr(expt)	Mr(calc)	Delta	Miss	Score	Rank	Peptide
29	485.27	968.52	968.46	0.06	0	26	1	FADENFVK
42	756.40	1510.78	1510.72	0.05	0	17	1	VFFDMTIGGQPAGR + Oxidation (M)
48	691.32	2070.95	2070.99	-0.04	0	(21)	2	IVMELYADVTPSTAENFR + Oxidation (M)
49	691.38	2071.12	2070.99	0.12	0	46	1	IVMELYADVTPSTAENFR + Oxidation (M)

Spot No. 17 Cytosolic malate dehydrogenase [Glycine max (Soybean)] TA43756_3847 **Mass:** 16551 **Total score:** 173 **Peptides matched:** 7

Query	Observed	Mr(expt)	Mr(calc)	Delta	Miss	Score	Rank	Peptide
18	362.23	722.44	722.40	0.05	0	33	1	NVSIYK
38	494.85	987.68	987.56	0.12	0	47	1	LTVQVSDVK
41	508.33	1014.64	1016.52	-1.88	0	(37)	1	EFAPSIPEK
42	509.33	1016.65	1016.52	0.13	0	46	1	EFAPSIPEK
61	550.50	1648.48	1648.99	-0.51	0	(44)	1	VLVVANPANTNALILK
62	825.56	1649.11	1648.99	0.12	0	47	1	VLVVANPANTNALILK
63	550.73	1649.15	1648.99	0.17	0	(45)	1	VLVVANPANTNALILK

Spot No. 18 20S proteasome beta subunit C-1 [Arabidopsis thaliana] TA46641_3847 **Mass:** 15278 **Total score:** 87 **Peptides matched:** 2

Query	Observed	Mr(expt)	Mr(calc)	Delta	Miss	Score	Rank	Peptide
42	530.36	1588.07	1588.86	-0.78	0	(25)	1	LGVQLQTIATDFQR
43	795.50	1588.98	1588.86	0.13	0	87	1	LGVQLQTIATDFQR

Spot No. 19 Gamma-glutamyl hydrolase precursor [Glycine max (Soybean)] TA43569_3847 **Mass:** 27513 **Total score:** 315 **Peptides matched:** 10

Query	Observed	Mr(expt)	Mr(calc)	Delta	Miss	Score	Rank	Peptide
13	353.22	704.43	705.38	-0.95	0	(18)	4	YAISPR
14	354.19	706.36	705.38	0.98	0	30	3	YAISPR
19	411.70	821.38	821.40	-0.02	0	34	1	FVESGGAR
42	737.38	1472.75	1472.77	-0.02	0	34	1	YPVTVNLWQPEK
52	785.89	1569.77	1569.78	-0.00	0	94	1	VTQSTANFFISEAR
53	524.27	1569.79	1569.78	0.01	0	(47)	2	VTQSTANFFISEAR
59	871.99	1741.96	1741.92	0.03	0	52	1	VIPLIYNESPENLNK
65	624.66	1870.95	1870.02	0.93	1	36	1	VIPLIYNESPENLNKK
69	648.31	1941.90	1941.93	-0.03	1	(34)	1	LSDFFEILATSEDRDGK
70	972.00	1941.98	1941.93	0.04	1	40	1	LSDFFEILATSEDRDGK

Spot No. 20 Cationic peroxidase 1, putative, expressed [Oryza sativa (japonica cultivar-group)] TA64044_3847 **Mass:** 25311 **Total score:** 136 **Peptides matched:** 5

Query	Observed	Mr(expt)	Mr(calc)	Delta	Miss	Score	Rank	Peptide
13	396.72	791.42	790.43	1.00	0	25	3	MGLLDVK + Oxidation (M)
29	555.28	1662.83	1663.82	-0.99	0	(21)	2	NGVLTSDQTLYNSPR
30	555.31	1662.90	1663.82	-0.92	0	(25)	1	NGVLTSDQTLYNSPR
31	832.93	1663.85	1663.82	0.03	0	110	1	NGVLTSDQTLYNSPR
32	833.42	1664.82	1663.82	1.00	0	(83)	1	NGVLTSDQTLYNSPR

Spot No. 21 Beta-amylase [Glycine max (Soybean)] TA44402_3847 **Mass:** 25349 **Total score:** 203 **Peptides matched:** 5

Query	Observed	Mr(expt)	Mr(calc)	Delta	Miss	Score	Rank	Peptide
25	435.31	868.60	868.44	0.15	0	19	2	SNFNIFK
26	463.31	924.60	925.50	-0.90	0	35	1	VAGENALPR
42	593.81	1185.61	1185.62	-0.01	0	(16)	2	LSMFGVTYLR
44	601.81	1201.61	1201.62	-0.01	0	71	1	LSMFGVTYLR + Oxidation (M)
62	813.42	1624.83	1624.82	0.01	0	79	1	YDATAYNQIILNAR

Spot No. 22 Beta-amylase [Glycine max (Soybean)] TA44402_3847 **Mass:** 24263 **Total score:** 391 **Peptides matched:** 11

Query	Observed	Mr(expt)	Mr(calc)	Delta	Miss	Score	Rank	Peptide
24	463.76	925.51	925.50	0.01	0	49	1	VAGENALPR
28	499.26	996.50	997.47	-0.97	0	29	1	SNGTYVTEK
34	593.80	1185.59	1185.62	-0.03	0	(37)	1	LSMFGVTYLR
35	601.81	1201.60	1201.62	-0.02	0	52	1	LSMFGVTYLR + Oxidation (M)
49	702.32	1402.62	1402.64	-0.02	0	61	1	YNDVPESTGFFK
54	761.34	1520.66	1520.69	-0.03	0	56	1	AGHPEWELPDDAGK
55	507.90	1520.69	1520.69	0.00	0	(37)	1	AGHPEWELPDDAGK
61	790.39	1578.77	1578.80	-0.03	0	59	1	LLNHGDQILDEANK
62	527.27	1578.80	1578.80	-0.00	0	(38)	1	LLNHGDQILDEANK
75	683.99	2048.93	2048.96	-0.02	0	86	1	VENHAAELTAGYYNLNDR
76	1025.49	2048.96	2048.96	0.01	0	(75)	1	VENHAAELTAGYYNLNDR

Spot No. 23 Stem 31 kDa glycoprotein precursor [Glycine max (Soybean)] TA41978_3847 **Mass:** 17882 **Total score:** 269 **Peptides matched:** 9

Query	Observed	Mr(expt)	Mr(calc)	Delta	Miss	Score	Rank	Peptide
18	382.24	762.48	762.46	0.01	0	35	1	LVSLGFK
21	403.26	804.50	804.49	0.02	0	36	3	IIFLSGR
33	487.28	972.54	972.52	0.02	0	63	1	QAVTEANLK
36	555.30	1108.58	1109.61	-1.03	0	(39)	1	GNAPALPETLK
37	555.82	1109.63	1109.61	0.02	0	47	1	GNAPALPETLK
50	680.83	1359.65	1359.66	-0.01	0	35	1	TVNQQAYFYAR
51	454.25	1359.73	1359.66	0.08	0	(29)	1	TVNQQAYFYAR
53	477.25	1428.74	1429.78	-1.04	1	20	2	TLDKQAVTEANLK
61	662.67	1985.00	1985.05	-0.05	1	33	2	LILKDPQDPSTPNAVSYK
